# *MAP2* – A Candidate Gene for Epilepsy, Developmental Delay and Behavioral Abnormalities in a Patient With Microdeletion 2q34

**DOI:** 10.3389/fgene.2018.00099

**Published:** 2018-03-26

**Authors:** Dominik S. Westphal, Stephanie Andres, Christine Makowski, Thomas Meitinger, Julia Hoefele

**Affiliations:** ^1^Institute of Human Genetics, Technical University of Munich, Munich, Germany; ^2^Institute of Human Genetics, Helmholtz Zentrum München, Neuherberg, Germany; ^3^Department of Pediatrics, Technical University of Munich, Munich, Germany

**Keywords:** 2q34 deletion, behavioral abnormalities, seizure, developmental delay, *MAP2*

## Abstract

**Introduction:** Microdeletions in the chromosomal region 2q34 and its neighboring regions lead to a phenotypic spectrum including autism, intellectual disability, and epilepsy. Up to now, only few affected patients have been reported. Therefore, the genetic pathogenesis is not completely understood. One of the most discussed candidate genes in this context is *MAP2*, a gene responsible for microtubule polymerization and neurite outgrowth.

**Materials and Methods:** We present a 4.5-year-old male patient with epilepsy, mild developmental delay, and behavioral abnormalities. SNP-Array analysis was performed to search for pathogenic copy number variations.

**Results:** SNP-Array analysis revealed a 1.5 Mb *de novo* microdeletion on the long arm of chromosome 2 (2q34). The identified microdeletion included the candidate genes *UNC80, LANCL1*, and most importantly *MAP2*.

**Discussion:** The reported microdeletion identified in this patient is the smallest one described in the literature so far spanning *MAP2* next to *UNC80* and *LANCL1*. In this context *MAP2* is the most important candidate gene concerning neuronal development and its function should be further examined.

## Introduction

A number of genes that are relevant for neurodevelopment and neuronal function are located in the chromosomal region 2q34. Examples for this are *LANCL1* (Online Mendelian Inheritance in Man, OMIM, **^∗^**602070), encoding for lanthionine synthetase C-like protein 1 that is involved in nerve growth factor (NGF)-induced nerve growth (Zhang et al., 2009), and *MAP2* (OMIM ^∗^157130), encoding for microtubule-associated protein-2, a neurosteroid-receptor mediating microtubule polymerization, which is also taking part in NGF-induced neurite outgrowth (Fontaine-Lenoir et al., 2006). In 2003 the role of chromosomal region 2q34 in the context of neurodevelopment was emphasized by a single case described by Pescucci et al. (2003). They reported a 14-year-old patient with a microdeletion 2q34 who showed autistic traits and Rett-like features. *MAP2* was postulated as one of the possible syndrome-inducing candidate genes amongst others (Pescucci et al., 2003). Up to now, a few additional cases were reported concerning this chromosomal region. One of the most recent publications described a 4.5-year-old girl with autistic traits, Rett-like features, and a microdeletion 2q33.3q34. The authors concluded that *NRP2, ADAM23, KLF7, CREB1, MAP2, UNC80*, and *LANCL1*, all taking part in neuronal function, are possible candidate genes for this phenotype (Jang et al., 2015). Furthermore, in a genome-wide linkage meta-analysis the chromosomal region 2q34 was proposed as susceptibility locus (non-parametric logarithm of odds score 3.43) for juvenile myoclonic epilepsy (Consortium et al., 2012). In conclusion, this region is of high interest regarding neuronal development and function although all relevant factors have not been elucidated so far.

We present a 4.5-year-old male patient with the smallest microdeletion 2q34 reported so far. One of the genes in this 1.5 Mb spanning region is the candidate gene *MAP2*. The patient’s clinical features include mild developmental delay, behavioral abnormalities as well as epilepsy.

## Materials and Methods

The study was approved by the local Ethics Committee of the Technical University of Munich and performed according to the standard of the Helsinki Declaration of 1975. Blood samples for molecular karyotyping were collected from the patient and the parents after written informed consent. Written informed consent concerning publication of this case report was also obtained from the parents of the participant.

DNA was extracted from peripheral blood using the Gentra Puregene Blood Kit (Qiagen, Hilden, Germany) according to the manufacturer’s instructions. DNA samples of the patient and his parents were analyzed using the single nucleotide polymorphism (SNP)-Array Affymetrix^®^ CytoScan^TM^ 750K Array (Affymetrix^®^ Inc., Santa Clara, CA, United States) with an average space of 4 kb between two oligonucleotides. Scanning was performed by the Affymetrix^®^ GeneChip Scanner 3000 7G (resolution 0.51–2.5 μm). The data analysis was conducted using the Affymetrix^®^ Chromosome Analysis Suite Software (ChAS), version 3.0, hg19.

## Case Report and Results

**FIGURE 1:**
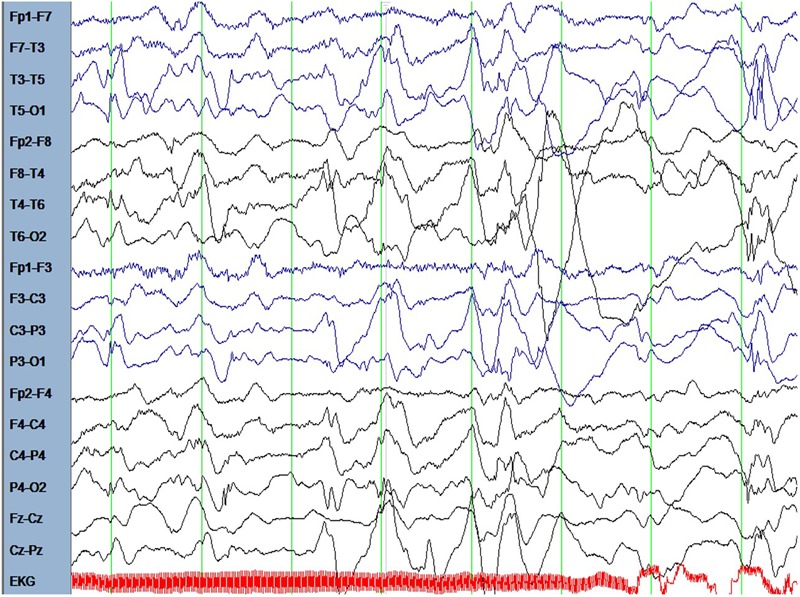
The EEG at the age of 6.5 months showed hypsarrhythmia.

**FIGURE 2:**
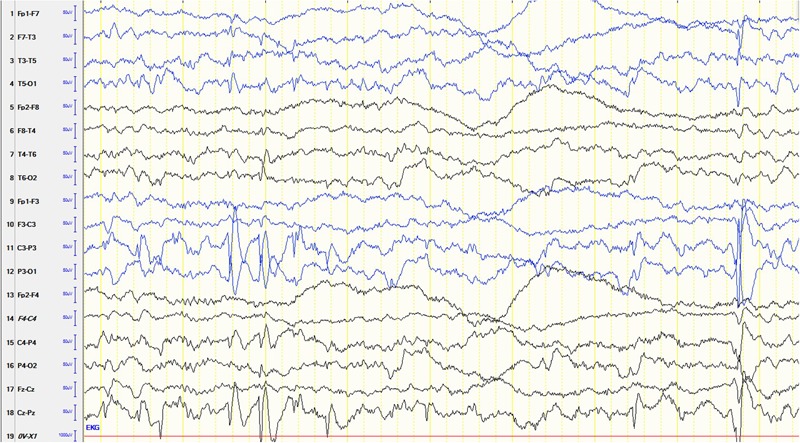
An EEG performed at the age of 3.5 years showed sharp waves located left centrotemporal.

The male patient is the first and only child of healthy parents. He was born at gestational age of 37 weeks. His birth measurements were as follows: birth weight 2730 g (-1.5 standard deviations, SD), birth length 49 cm (-0.68 SD), head circumference at birth 34 cm (-0.43 SD). There were no dysmorphological signs seen at birth. From the beginning, the boy had difficulties falling asleep and suffered from insomnia until the age of 15 months. He was presented at the children’s hospital at the age of 6.5 months because of weight loss and recurrent emesis. One month later the mother noted several episodes of typical Blitz-Nick-Salaam (BNS) seizures. An electroencephalogram (EEG) was performed and showed hypsarrhythmia (**Figure 1**). Consequently, the patient was diagnosed as having West syndrome. A medication with vitamin B6 and sulthiame was started. After 5 days the seizures disappeared. A motoric developmental delay had also been noted before seizure onset which worsened in the course of the disease. The patient was not capable of rolling from supine to prone position anymore and showed partly limited head control when erected from supine position. A magnetic resonance imaging (MRI) of the brain performed at the age of 9 months was normal. Vitamin B6 was stopped at that time. At the age of 14 months his developmental skills were at the level of a 10-month-old infant. A study of his behavior showed that he was seeking for attention and stimuli to stabilize and regulate himself even when being tired and exhausted. Sulthiame was reduced at the age of 19 months and stopped at the age of 22 months as there were no more seizures and the EEG was normal. At the age of 26 months his mother described a behavior that was in line with a lack of impulse control. He also had problems in closeness-distance regulation, showed short spans of concentration, and was distracted easily. In an EEG performed at the age of 3.5 years rolandic spikes located left centrotemporally were detected (**Figure 2**). Sleep activation was seen in another EEG without clinical seizures. Therefore, a medication with sulthiame was started again. The pathological findings in the EEG improved in the further course. A MRI of the brain reconfirmed that there were no pathological signs present. Although he improved his psychomotor skills and was able to run and speak, his fine motor skills at the age of 4.5 years were comparable to a 2.5-year-old child. At that age he was presented at our institute for genetic counseling.

**FIGURE 3:**
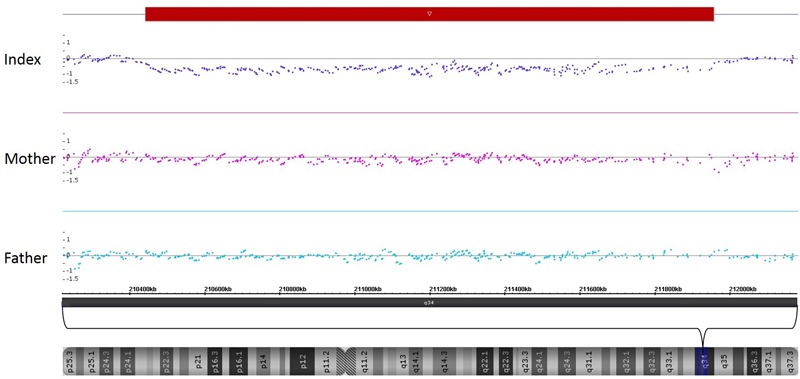
SNP-Array-analysis of the patient identified a microdeletion in the chromosomal region 2q34 (red bar, genome position: chr2:210,440,530-211,955,369, GRCh37/hg19). Comparison with the parents’ results revealed the *de novo* status of this microdeletion.

**FIGURE 4:**
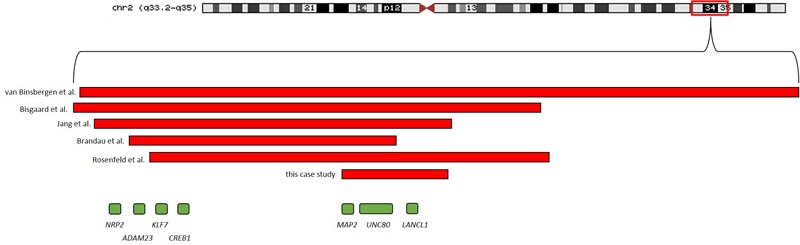
Microdeletions including *MAP2* in previous reported patients as well as the microdeletion identified in the patient of this study are shown.

SNP-Array analysis revealed a 1.5 Mb microdeletion in the chromosomal region 2q34 including eight OMIM genes (*MAP2, UNC80, RPE, KANSL1L, ACADL, MYL1, LANCL1*, and *CPS1*) out of nine Reference Sequence (RefSeq) genes (genome position: chr2:210,440,530-211,955,369, GRCh37/hg19). Three transcript variants of *MAP2* (NM_002374.3, NM_031847.2, and NM_031845.2) were completely included in this microdeletion. One transcript variant of *MAP2* (NM_001039538.1) was deleted except for the two first exons. Subsequent SNP-Array analysis of the parents revealed the *de novo* status of the patient’s microdeletion (**Figure 3**). A comparison of the identified microdeletion 2q34 with previously reported microdeletions in the chromosomal region 2q33.2-q35 including *MAP2* (**Figure 4**) showed that the microdeletion in our patient is the shortest reported so far. *MAP2, UNC80* and *LANCL1* are amongst the proposed candidate genes by Jang et al. (2015) for the phenotype in patients with a microdeletion 2q33.3q34 (**Figure 4**).

## Discussion

There are several genes located in the chromosomal region 2q34 that have a part in neuronal development and function. This region has already been associated with juvenile myoclonic epilepsy and Alzheimer’s disease by linkage analysis (Scott et al., 2003; Consortium et al., 2012). Up to now, only a few cases have been reported that describe neurological abnormalities due to a loss of this critical region. Thus, especially concerning developmental delay and autistic traits in behavior, there is more than one candidate gene to be expected. One of the most promising genes causing this symptomatic is *MAP2* (Pescucci et al., 2003; Mukaetova-Ladinska et al., 2004; Rosenfeld et al., 2010; van Binsbergen et al., 2014; Jang et al., 2015). The case of our patient affected by developmental delay, epilepsy, neurocognitive, and behavioral abnormalities is fitting to the hypothesis that *MAP2* is one of the candidate genes, as our patient’s microdeletion is the smallest one described in the literature so far spanning *MAP2* amongst other genes. Although one transcript is not completely deleted it can be expected that there is a loss of function of *MAP2* because only the first two exons remain.

*MAP2* is a neurosteroid receptor important for the microtubule polymerization in the outgrowth of neurites (Fontaine-Lenoir et al., 2006). The reason for *MAP2* being the most likely candidate gene responsible for the patient’s phenotype is its high probability of loss of function intolerance (pLI) generated by >120,000 control alleles of the Exome Aggregation Consortium (ExAC) browser. It’s the only pLI of the deleted genes in our patient that reaches the amount of 1.0. Furthermore, in ExAC there is no deletion listed concerning *MAP2*. The pLI was calculated as previously described (Lek et al., 2016). In 1997 evidence was already present that *MAP2* expression is reduced in patients with Rett syndrome while being upregulated in Down syndrome (Kaufmann et al., 1997). Mukaetova-Ladinska et al. (2004) examined postmortem the *MAP2* expression in the dorsolateral prefrontal cortex in two individuals with autistic features, one with recorded epileptic seizures from birth. They reported a depletion of the *MAP2* expression in neurons and dendrites, particular in the cortical domain, as well as changes in the cytoarchitecture of the cortex (Mukaetova-Ladinska et al., 2004). Van Binsbergen et al. (2014) identified a 14.79 Mb *de novo* deletion on 2q33.2q35 in a fetus with brain developmental anomalies (i.e., delayed gyral formation, neuronal heterotopia of the white matter, small cerebellum) amongst other syndromic features and emphasized *MAP2*’s role in neurodevelopment. Our patient showed no abnormalities in the MRI of the brain consistent with other *MAP2*-deficient patients reported so far (Bisgaard et al., 2006; Brandau et al., 2008; Jang et al., 2015). Also noteworthy is a recent publication in Nature in which out of 8,361 reported candidate *de novo* non-sense variants in patients with developmental disorder four were located in *MAP2* (Deciphering Developmental Disorders Study, 2017).

Another two candidate genes for the 2q34 phenotype, spanned by the microdeletion in our patient, are *UNC80* and *LANCL1*. These had also already been proposed as candidate genes by Jang et al. (2015), *UNC80* seems to be involved in the Ca^2+^ dependent excitability of neurons (Lu et al., 2010). It has been associated with the clinical phenotype of autosomal-recessive inherited infantile hypotonia with psychomotor retardation and characteristic facies 2 (IHPRF2; OMIM ^∗^612636). With a pLI of 0.14 it is unlikely that a possible haploinsufficiency of this gene might contribute to the phenotype of our patient. However, it should be noted that the symptoms resulting from a microdeletion of a single gene are not comparable with the symptoms caused by a contiguous gene syndrome. Therefore, *UNC80* might also contribute to the phenotype of our patient. The other candidate gene already mentioned is *LANCL1*, taking part in NGF-induced nerve growth (Zhang et al., 2009). A more recent publication by Huang et al. (2014) showed that the transcribed protein LANCL1 is important for neuronal protection from reactive oxygen species (ROS). The authors reported postnatal neuronal degeneration in *LANCL1* knockout mice (*LANCL1* -/-) (Huang et al., 2014). Like in the case of *UNC80* it is not very likely that a heterozygous loss of *LANCL1* leads to clinical symptoms because of a pLI value of 0. But taken together it has to be emphasized that this region and their genes seems to be important for the proper neuronal development and function.

In the latest publication concerning microdeletion 2q34 and 2q33.3, respectively, the authors additionally mentioned *CREB1, KLF7, ADAM23*, and *NRP2* (Jang et al., 2015). These genes are not spanned by the microdeletion in our patient. Their role in contributing to the phenotype of patients with a microdeletion 2q33q34 remains still to be elucidated as the clinical symptoms of our patient seem to be considerably more moderate. There are a few patients whose microdeletion do not affect the now discussed region in our patient, like the microdeletion spanning *ERBB4* that is located more distally in 2q34 in a patient with intellectual disability and hyperactivity (Kasnauskiene et al., 2013) or patients with the proximal microdeletion 2q32q33 and severe mental retardation (Van Buggenhout et al., 2005).

## Conclusion

The 1.5 Mb microdeletion on 2q34 in our patient is the shortest microdeletion spanning *MAP2*, a top candidate gene responsible for autism and developmental delay, reported so far. Although the phenotype composed of epilepsy, developmental delay, and behavioral abnormalities was mild in our patient in contrast to other patients with a microdeletion on 2q33q34, our case might help to elucidate the contribution of *MAP2* to this exceedingly interesting region in context of neuronal development and function. The patient has the unusual presentation of a BNS epilepsy with rapid response to treatment with pyridoxine and sulthiame and an EEG with rolandic spikes 3 years later without having seizures.

## Author Contributions

DW: wrote the manuscript and did the genetic counseling. SA: performed the SNP-Array analysis. CM: pediatrician who performed the EEG and did the medical treatment of the patient. TM: head of the institute, provided the material, methods, and education. JH: supervised the genetic counseling and the whole process of the making of the manuscript.

## Conflict of Interest Statement

The authors declare that the research was conducted in the absence of any commercial or financial relationships that could be construed as a potential conflict of interest.
